# Complementary Health Approach Utilization Among U.S. Adults With Attention-Deficit/Hyperactivity Disorder: A Nationally Representative Survey

**DOI:** 10.7759/cureus.112286

**Published:** 2026-07-08

**Authors:** Jackson H Wheatley, Thomas Motyka, Kyle Agostini

**Affiliations:** 1 Osteopathic Medicine/Internal Medicine, Campbell University School of Osteopathic Medicine, Buies Creek, USA

**Keywords:** adult mental health, attention-deficit/hyperactivity disorder, community mental health, complementary health approaches, health services utilization

## Abstract

Introduction

Attention-deficit/hyperactivity disorder (ADHD) frequently persists into adulthood and is associated with substantial functional impairment and unmet mental health needs. Complementary health approaches (CHAs) are commonly used by individuals with chronic mental health conditions; however, nationally representative data describing CHA utilization among U.S. adults with ADHD are limited. This study aims to examine patterns of CHA utilization among U.S. adults with ADHD and evaluate whether ADHD diagnosis was independently associated with CHA use.

Methods

We analyzed data from a nationally representative, quota-matched, cross-sectional survey of U.S. adults conducted in August 2024 (N = 3,022). ADHD status was based on self-reported clinician diagnosis. Participants reported CHA use within the past 12 months, as well as favorable expectations and experiences. CHAs were categorized as nutritional, psychological, physical, combined psychological and physical, or medical system-based-approaches. Logistic regression models estimated unadjusted and adjusted odds ratios (ORs) with 95% confidence intervals (CIs), controlling for demographic and socioeconomic covariates.

Results

Adults with ADHD (n = 474; 15.7%) were more likely to report use of any CHA compared with adults without ADHD in both unadjusted (OR 2.14, 95% CI 1.20-3.81) and adjusted analyses (adjusted OR 3.21, 95% CI 1.76-5.84). ADHD diagnosis was independently associated with higher utilization of physical, psychological, and combined psychological and physical approaches. No significant adjusted association was observed for nutritional approaches. Favorable expectations and experiences did not consistently differ by ADHD status after adjustment.

Conclusions

U.S. adults with ADHD demonstrate higher utilization of complementary health approaches, particularly those addressing psychological and physical domains. Differences in utilization were not consistently explained by more favorable expectations or experiences. These findings highlight patterns of complementary health use among adults with ADHD that may inform comprehensive, patient-centered-care.

## Introduction

Attention deficit/hyperactivity disorder (ADHD) is a chronic neurodevelopmental condition that persists into adulthood in a substantial proportion of affected individuals and is associated with significant functional, psychological, and social impairment. In the United States, approximately 4-5% of adults meet diagnostic criteria for ADHD, and many experience persistent symptoms that interfere with occupational functioning, interpersonal relationships, and overall quality of life [1,2]. Adults with ADHD frequently experience co-occurring psychiatric symptoms and diagnoses, including anxiety, depression, and substance use disorders, although diagnostic overlap and misclassification may contribute to some observed associations [3,4]. These challenges may complicate long-term management and increase demand for mental health services.

Evidence-based treatments for adult ADHD, including stimulant and non-stimulant pharmacotherapy and structured psychosocial interventions, are effective for many individuals [5]. However, treatment access, adherence, and satisfaction remain ongoing challenges. Barriers include limited availability of specialty providers, cost and insurance constraints, medication side effects, stigma-related-concerns, and variability in treatment response [5]. As a result, many adults with ADHD report unmet treatment needs and pursue additional or alternative strategies to manage symptoms and associated distress [6].

Complementary health approaches (CHAs), defined as non-mainstream practices used alongside conventional medical or mental health care, represent one such strategy [7]. CHAs encompass a broad range of modalities, including mind-body practices (e.g., meditation, yoga), manual therapies (e.g., chiropractic care, massage), biologically based approaches (e.g., dietary supplements, specialized diets), and spiritually-oriented practices [7]. In mental health populations, CHA use has been associated with efforts to address residual symptoms, reduce stress, enhance well-being, and increase involvement in treatment decisions [8].

Prior research suggests that individuals with chronic mental health conditions are more likely to engage in CHAs than the general population, typically as adjuncts rather than replacements for conventional care [9,10]. However, studies examining CHA use in ADHD populations have largely focused on children and adolescents, relied on small clinical samples, or emphasized efficacy rather than utilization patterns [11,12]. Population-level data describing CHA utilization among adults with ADHD remain limited, particularly within community based settings.

Understanding CHA utilization among adults with ADHD has important implications for mental health systems. Use of complementary approaches may be associated with unmet clinical needs, barriers to conventional services, dissatisfaction with treatment, incorrect underlying diagnosis, or attempts at self-management. Unrecognized CHA use may contribute to fragmented care or limit opportunities for informed counseling [13]. Improved awareness of these patterns may support more coordinated and patient-centered-care.

Despite the clinical and policy relevance of these issues, nationally representative data examining CHA utilization among U.S. adults with ADHD remain sparse. Previous national surveys have often grouped ADHD with other psychiatric conditions or aggregated complementary practices into broad categories, limiting disorder-specific interpretation [14,15]. Few studies have evaluated CHA use among adults with ADHD while accounting for demographic and socioeconomic factors known to influence mental health service utilization.

The present study addresses these gaps by examining the association between adult ADHD diagnosis and complementary health approach utilization in a nationally representative sample of U.S. adults. Using data collected via the Qualtrics XM platform [16], we aimed to characterize patterns of CHA use among adults with ADHD and assess whether ADHD diagnosis is independently associated with utilization after controlling for demographic and socioeconomic variables.

## Materials and methods

Study design and data source

This study involved a secondary analysis of data from a cross-sectional, quota-controlled online survey of U.S. adults conducted in August 2024. The survey was administered by Qualtrics, LLC, using a large national opt-in panel of individuals who voluntarily enroll to participate in surveys [16]. Panel enrollment was not based on ADHD status or any other health diagnosis. Participants received modest compensation through the panel provider for survey completion. Demographic quota targets were applied during recruitment to approximate U.S. Census distributions for age, sex, race and ethnicity, geographic region, and educational attainment. The survey assessed health conditions, healthcare utilization, and use of complementary health approaches (CHAs) among U.S. adults. Participants were eligible if they were aged 18 years or older, resided in the United States as verified by IP-based geolocation, and could complete the survey in English. All participants provided electronic informed consent. Only participants who completed the full survey and passed embedded data quality checks were included in the analytic sample. The dataset used in this study is not publicly available. Data was obtained through a Qualtrics-administered national opt-in panel as part of a commissioned survey and are accessible only to the investigative team under the terms of the panel provider agreement. To our knowledge, this dataset has not been previously published elsewhere. The objectives of this study were to characterize patterns of complementary health approach utilization among U.S. adults with ADHD, examine whether ADHD diagnosis was independently associated with CHA use after adjustment for demographic and socioeconomic factors, and evaluate favorable expectations and experiences related to CHA use.

Ethical considerations

The dataset used for this analysis was fully de-identified prior to receipt by the investigators. The original data collection protocol was reviewed by the Institutional Review Board at Campbell University and determined not to constitute human subjects research as defined under the Code of Federal Regulations (45 CFR 46.102) because it used fully de-identified secondary data. Additional institutional review board approval was not required for this analysis.

Measures

Attention-Deficit/Hyperactivity Disorder Status

ADHD status was determined by self-report. Participants were classified as having ADHD if they answered affirmatively to the question, “Have you ever been diagnosed with attention-deficit/hyperactivity disorder (ADHD) by a healthcare professional?” Participants who responded “no” served as the comparison group. This approach is consistent with prior epidemiologic studies of ADHD prevalence and healthcare utilization in large population-based samples.

Complementary Health Approach Utilization

Use of CHAs was assessed by asking participants whether they had used specific non-mainstream health practices within the past 12 months. Individual modalities were grouped into five analytic categories based on conceptual similarity and prior classification frameworks, as detailed in Table 1 [7].

**Table 1:** Analytic grouping of complementary health approaches assessed in the survey Complementary health approaches (CHA) were grouped for analytic clarity and descriptive purposes. Categories reflect shared conceptual features but do not imply similarity in clinical effectiveness, mechanism of action, or evidence base. Classification was used solely to examine patterns of utilization within a community mental health framework. The nutritional approaches category includes commonly used self-management supplements such as omega-3 fatty acids and other dietary supplements, as well as vitamin and mineral preparations (e.g., zinc and magnesium) when reported by participants. Homeopathic preparations were classified as nutritional approaches based on analytic grouping informed by National Center for Complementary and Integrative Health (NCCIH) complementary health classification frameworks and for consistency with the survey instrument. Homeopathic preparations were grouped within the nutritional approaches category for analytic purposes because they are typically self-administered ingestible products used outside conventional medical care and were assessed alongside dietary supplements and related consumer-directed products in the survey instrument. This classification was used solely for analytic consistency and does not imply that homeopathy shares the same mechanism of action, evidence base, or theoretical framework as nutritional interventions.

Analytic CHA category	Modalities included in analysis
Nutritional approaches	Use of vitamins or minerals; non–vitamin or non–mineral dietary supplements; consultation with an herbalist; non-prescription dietary regimens; homeopathic preparations
Psychological approaches	Spiritual healing or prayer practices; mindfulness-based practices; shamanic or ritual healing experiences; therapist-guided hypnosis; self-directed hypnosis
Physical approaches	Chiropractic care; osteopathic manipulative treatment; massage therapy; light-, magnetic-, or electrical-based therapies not delivered as physical therapy; other nonconventional therapeutic devices
Combined psychological and physical approaches	Breathing exercises; progressive muscle relaxation; meditation practices; biofeedback; guided imagery; acupuncture; yoga; tai chi or qigong; nonconventional movement-based therapies; expressive therapies (e.g., art, music, or dance therapy)
Medical system–based approaches	Engagement with traditional medical systems (e.g., Ayurveda, Traditional Chinese Medicine); naturopathic care

For the primary analysis, CHA utilization was operationalized as a binary outcome indicating at least one CHA use in the past 12 months. Secondary analyses examined utilization across the five categories. These classifications were used solely for analytic purposes and do not imply equivalence in therapeutic mechanisms, evidence base, or clinical indications.

Demographic and Socioeconomic Covariates

Demographic variables included age (18-34, 35-49, 50-64, 65+ years), sex at birth, race and ethnicity (White non-Hispanic, Black non-Hispanic, Hispanic, other subjects), educational attainment (less than college vs. some college or higher), employment status (not working, part-time, full-time), annual household income (self-reported), health insurance status (insured vs. uninsured), geographic region (Northeast, Midwest, South, West), and residential setting (city, town, village, countryside). These variables were selected a priori based on prior associations with mental health service utilization and CHA use.

Data quality and missing data

Survey responses were screened using embedded attention-check items. Respondents who failed these checks were excluded. Missing data was minimal across most variables due to fixed-choice response formats. Household income was the only variable with notable nonresponse; participants with missing income data were retained using available case methods. No imputation procedures were applied.

Statistical analysis

Descriptive statistics summarized participant characteristics and CHA utilization patterns by ADHD status. Pearson Chi-square tests assessed bivariate associations. Binary logistic regression models estimated unadjusted and adjusted odds ratios (ORs) with 95% confidence intervals (CIs) for associations between ADHD status and complementary health approach utilization. Unadjusted odds ratios were derived from bivariate analyses of ADHD status and CHA outcomes, with statistical significance assessed using Pearson chi-square tests. The primary multivariable model examined any use of CHA, adjusting for demographic and socioeconomic covariates. Secondary models examined utilization across analytic CHA categories. Covariates were entered simultaneously. Multicollinearity was assessed using variance inflation factors (VIFs), with values greater than five considered indicative of potentially problematic multicollinearity. Model fit was evaluated descriptively through review of logistic regression output and diagnostic statistics; formal goodness-of-fit testing was not performed. All analyses were conducted using IBM SPSS Statistics, version 26.0 (IBM Corp., Armonk, NY, USA). Statistical significance was defined as two-sided p < 0.05.

## Results

Sample characteristics

The analytic sample consisted of 3,022 adults, of whom 474 (15.7%) reported a clinician diagnosis of attention- deficit/hyperactivity disorder (ADHD). Demographic and socioeconomic characteristics stratified by ADHD status are presented in Table 2. Compared with participants without ADHD, adults with ADHD were younger, with nearly half aged 18-34 years, and were less likely to be aged 65 years or older (p<.001). Participants with ADHD were also more likely to report lower educational attainment, lower household income, and differences in employment status relative to those without ADHD (all p<.001). Significant differences were observed by race, ethnicity, and geographic region, whereas sex at birth, health insurance status, and residential setting did not differ significantly by ADHD status. These variables were included as covariates in adjusted analyses.

**Table 2:** Demographic and socioeconomic characteristics of participants by ADHD status Values are presented as n (%). Percentages are column percentages. P-values were calculated using Pearson Chi-square tests. ADHD: Attention-deficit/hyperactivity disorder.

Characteristic	Non-ADHD (n = 2,548)	ADHD (n = 474)	p-value
Age category			< .001
18–34 years	663 (26.0%)	227 (47.9%)	
35–49 years	587 (23.0%)	161 (34.0%)	
50–64 years	692 (27.2%)	69 (14.6%)	
≥65 years	606 (23.8%)	17 (3.6%)	
Sex at birth			0.48
Male	1,251 (49.1%)	246 (51.9%)	
Female	1,297 (50.9%)	228 (48.1%)	
Race and ethnicity			0.03
White, non-Hispanic	1,545 (60.6%)	306 (64.6%)	
Black, non-Hispanic	288 (11.3%)	37 (7.8%)	
Hispanic	454 (17.8%)	94 (19.8%)	
Other	261 (10.3%)	37 (7.8%)	
Educational attainment			< .001
Less than college	916 (35.9%)	221 (46.6%)	
Some college or higher	1,632 (64.1%)	253 (53.4%)	
Employment status			< .001
Not working	1,377 (54.0%)	202 (42.6%)	
Part-time	329 (12.9%)	86 (18.1%)	
Full-time	842 (33.1%)	186 (39.2%)	
Household income			< .001
Below poverty level	677 (26.6%)	192 (40.5%)	
Near poverty	293 (11.5%)	51 (10.8%)	
Above poverty	1,578 (61.9%)	231 (48.7%)	
Health insurance status			0.14
Uninsured	1,540 (60.4%)	304 (64.1%)	
Insured	1,008 (39.6%)	170 (35.9%)	
Geographic region			0.02
Northeast	445 (17.5%)	70 (14.8%)	
Midwest	517 (20.3%)	123 (26.0%)	
South	994 (39.0%)	165 (34.8%)	
West	592 (23.2%)	116 (24.5%)	
Residential setting			0.4
City	1,326 (52.0%)	239 (50.4%)	
Town	752 (29.5%)	135 (28.5%)	
Village or countryside	470 (18.4%)	100 (21.1%)	

Classification of complementary health approaches

Complementary health approaches (CHAs) assessed in the survey were grouped into five analytic categories for descriptive and inferential analyses: nutritional approaches, psychological approaches, physical approaches, combined psychological and physical approaches, and medical system-based approaches. The specific modalities included within each category are summarized in Table 1. These groupings were used to examine overall patterns of CHA utilization and to support category-level regression analyses.

Complementary health approach utilization

Overall CHA use was common in the sample, with higher prevalence among adults with ADHD compared with those without ADHD. Associations between ADHD diagnosis and CHA utilization are shown in Table 3.

**Table 3:** Association between adult ADHD diagnosis and CHA utilization Odds ratios (ORs) were estimated using logistic regression models. The outcome variable was any complementary health approach (CHA) use in the past 12 months (yes/no). Adults without attention-deficit/hyperactivity disorder (ADHD) served as the reference group. Adjusted models controlled for age, sex, race and ethnicity, educational attainment, employment status, household income, health insurance status, geographic region, and residential setting.

Complementary health approach category	Unadjusted OR (95% CI)	Adjusted OR (95% CI)
Any complementary health approach use	2.14 (1.20–3.81)	3.21 (1.76–5.84)
Nutritional approaches	0.90 (0.71–1.15)	1.26 (0.97–1.64)
Psychological approaches	1.13 (0.90–1.40)	1.34 (1.06–1.70)
Physical approaches	1.94 (1.59–2.39)	1.86 (1.50–2.31)
Combined psychological and physical approaches	2.57 (1.96–3.35)	2.17 (1.64–2.88)
Medical system–based approaches	1.79 (1.37–2.32)	1.73 (1.30–2.30)

In unadjusted analyses, adults with ADHD had significantly higher odds of reporting any CHA use within the past 12 months compared with adults without ADHD (OR = 2.14, 95% CI = 1.20-3.81). This association remained significant and increased in magnitude after adjustment for demographic and socioeconomic covariates (adjusted OR = 3.21, 95% CI = 1.76-5.84). At the category level, ADHD diagnosis was significantly associated with higher utilization of physical approaches and combined psychological and physical approaches in both unadjusted and adjusted models. Adults with ADHD had higher adjusted odds of using physical approaches (adjusted OR = 1.86, 95% CI = 1.50-2.31) and combined psychological and physical approaches (adjusted OR = 2.17, 95% CI = 1.64-2.88) compared with adults without ADHD. Utilization of psychological approaches was also more likely among adults with ADHD after adjustment (adjusted OR = 1.34, 95% CI = 1.06-1.70).

No statistically significant association was observed between ADHD diagnosis and nutritional approach utilization after adjustment for covariates (adjusted OR = 1.26, 95% CI = 0.97-1.64). Adults with ADHD demonstrated higher odds of medical system-based CHA use, including traditional medical systems and naturopathy, in both unadjusted and adjusted analyses (adjusted OR = 1.73, 95% CI = 1.30-2.30).

Favorable expectations of complementary health approaches

Associations between ADHD diagnosis and favorable expectations regarding CHA use are presented in Table 4. In unadjusted analyses, adults with ADHD did not differ significantly from those without ADHD in favorable expectations of any CHA. After adjustment for demographic and socioeconomic factors, ADHD diagnosis was not significantly associated with favorable expectations of overall CHA use.

**Table 4:** Association between adult ADHD diagnosis and favorable expectations of complementary health approaches Odds ratios (ORs) were estimated using logistic regression models. The outcome variable was favorable expectation of complementary health approaches (yes/no). Adults without attention-deficit/hyperactivity disorder (ADHD) served as the reference group. Adjusted models controlled for age, sex, race and ethnicity, educational attainment, employment status, household income, health insurance status, geographic region, and residential setting.

Complementary health approach category	Unadjusted OR (95% CI)	Adjusted OR (95% CI)
Favorable expectation of any CHA	1.16 (0.95–1.42)	1.14 (0.92–1.41)
Nutritional approaches	0.92 (0.75–1.12)	0.90 (0.73–1.11)
Psychological approaches	1.31 (1.06–1.61)	1.24 (0.99–1.54)
Physical approaches	1.14 (0.94–1.39)	1.14 (0.92–1.40)
Combined psychological and physical approaches	1.14 (0.93–1.39)	1.15 (0.93–1.42)
Medical system–based approaches	0.91 (0.75–1.12)	0.93 (0.75–1.16)

At the category level, adults with ADHD demonstrated modestly higher odds of favorable expectations for psychological approaches in unadjusted analyses; however, this association did not reach statistical significance in adjusted models (adjusted OR = 1.24, 95% CI = 0.99-1.54). No significant adjusted associations were observed for nutritional, physical, combined psychological and physical, or medical system-based approaches.

Favorable experiences with complementary health approaches

Associations between ADHD diagnosis and favorable experiences with CHAs among respondents reporting prior use are shown in Table 5. In unadjusted analyses, adults with ADHD had lower odds of reporting favorable experiences with any CHA compared with adults without ADHD (OR = 0.65, 95% CI = 0.45-0.95). This association was attenuated and no longer statistically significant after adjustment for covariates (adjusted OR = 0.82, 95% CI = 0.55-1.22).

**Table 5:** Association between adult ADHD diagnosis and favorable experiences with complementary health approaches Odds ratios (ORs) were estimated using logistic regression models. The outcome variable was favorable experience with complementary health approaches (yes/no) among respondents reporting prior use. Adults without ADHD served as the reference group. Adjusted models controlled for age, sex, race and ethnicity, educational attainment, employment status, household income, health insurance status, geographic region, and residential setting.

Complementary health approach category	Unadjusted OR (95% CI)	Adjusted OR (95% CI)
Favorable experience with any CHA	0.65 (0.45–0.95)	0.82 (0.55–1.22)
Nutritional approaches	0.55 (0.40–0.76)	0.78 (0.55–1.09)
Psychological approaches	0.51 (0.35–0.74)	0.70 (0.47–1.04)
Physical approaches	0.61 (0.38–0.98)	0.77 (0.45–1.29)
Combined psychological and physical approaches	0.53 (0.36–0.79)	0.63 (0.41–0.96)
Medical system–based approaches	1.08 (0.57–2.07)	1.18 (0.58–2.42)

Similarly, lower odds of favorable experiences among adults with ADHD were observed across several CHA categories in unadjusted analyses, including nutritional, psychological, physical, and combined psychological and physical approaches. After adjustment, none of these category-level associations remained statistically significant, although point estimates continued to suggest a lower likelihood of favorable experiences among adults with ADHD. No significant associations were observed between ADHD diagnosis and favorable experiences with medical system-based CHAs in either unadjusted or adjusted models.

## Discussion

Summary of key findings

This nationally representative study examined patterns of complementary health approach (CHA) utilization among U.S. adults with attention deficit/hyperactivity disorder (ADHD) within a community-mental-health-context. Several key findings emerged. First, adults with ADHD were significantly more likely to report use of any CHA within the past 12 months compared with adults without ADHD, even after adjustment for demographic and socioeconomic factors. This association was robust and greater in magnitude in adjusted models, underscoring that increased CHA utilization among adults with ADHD is not solely attributable to sociodemographic differences.

Second, the strength of association between ADHD diagnosis and CHA use varied by analytic category. Utilization was most strongly associated with combined psychological and physical approaches and with physical approaches, followed by psychological approaches. In contrast, nutritional approaches were not significantly associated with ADHD diagnosis after adjustment. Third, although adults with ADHD were more likely to use CHAs, this increased utilization was not accompanied by consistently higher favorable expectations or more positive experiences. Exploratory analyses demonstrated that favorable expectations were largely similar between adults with and without ADHD, and that favorable experiences were comparable or, in some cases, less likely among adults with ADHD after accounting for covariates. Taken together, these findings suggest that increased CHA use among adults with ADHD reflects patterns of care-seeking behavior rather than uniformly positive subjective appraisal.

Complementary health approach utilization in adults with ADHD

The finding that adults with ADHD demonstrate substantially higher utilization of complementary health approaches aligns with broader literature showing increased use of non-mainstream health practices among individuals with chronic mental health conditions [9,10,17-19]. Adults with ADHD frequently experience persistent symptoms into adulthood, high rates of psychiatric comorbidity, and functional impairments across occupational, social, and emotional domains [3,12,20]. These factors may contribute to a heightened likelihood of seeking adjunctive or self-directed strategies alongside conventional mental health care, although the specific role of treatment access, treatment adequacy, and medication use cannot be determined from the present data [21-23].

Importantly, increased CHA utilization should not be interpreted as rejection of evidence-based treatments. Prior research suggests that complementary health approaches are most often used alongside, rather than in place of, conventional medical or mental health services [23-25]. For adults with ADHD, CHA use may reflect attempts to address residual symptoms, manage stress or emotional dysregulation, cope with medication side effects, or exert greater autonomy over health-related decision-making [26,27]. These motivations are particularly relevant in community mental health settings, where access to specialty ADHD care may be limited and continuity of care fragmented.

The magnitude of the association between ADHD diagnosis and CHA utilization observed in this study is notable compared with prior population-based estimates of Complementary and Alternative Medicine (CAM) use in psychiatric populations [10,21,23]. Even after adjustment for multiple socioeconomic indicators known to influence healthcare access and utilization, adults with ADHD remained significantly more likely to engage with CHAs. This suggests that ADHD itself, rather than social position alone, is associated with distinct patterns of health-seeking-behavior. From a community mental health perspective, this finding underscores the importance of recognizing CHA use as a common component of real-world care among adults with ADHD.

Interpretation of category-level patterns

Examination of CHA utilization by analytic category provides additional insight into how adults with ADHD engage with complementary approaches. The strongest associations were observed for combined psychological and physical approaches, as well as for physical approaches alone [13,25,27]. These categories include practices such as meditation, biofeedback, yoga, massage therapy, and chiropractic care, which may be perceived as addressing both psychological distress and somatic symptoms. Adults with ADHD often report heightened stress, sleep disturbances, and bodily tension and may therefore be drawn to modalities that emphasize relaxation, embodied regulation, or immediate experiential effects.

Psychological approaches, including mindfulness-based and spiritually-oriented practices, were also more commonly used among adults with ADHD after adjustment [13,21,28]. This finding is consistent with literature describing elevated psychological distress and emotion regulation difficulties in this population, which may prompt engagement with practices aimed at improving attentional control, stress management, or emotional well-being. In contrast, nutritional approaches were not independently associated with ADHD diagnosis after controlling for covariates, suggesting that use of supplements, special diets, or related strategies may be driven more strongly by demographic or cultural factors than by ADHD status per se [11,21,25].

Medical system-based approaches, including traditional medical systems and naturopathy, were used less frequently overall but remained more common among adults with ADHD [21,26,28]. Engagement with these systems may reflect broader dissatisfaction with conventional care or a desire for more holistic or individualized treatment frameworks. However, the relatively modest effect sizes observed in this category suggest that such approaches constitute a smaller component of CHA utilization compared with more accessible or widely available practices. Collectively, these category-level patterns highlight the heterogeneity of CHA use and underscore the importance of examining utilization beyond a single aggregated measure.

Favorable expectations and experiences: a secondary perspective

Although adults with ADHD were significantly more likely to utilize CHAs, this increased use was not accompanied by consistently higher favorable expectations or more positive experiences [21-23]. In adjusted analyses, favorable expectations were largely similar between adults with and without ADHD, and most category-level-associations did not reach statistical significance. These findings suggest that higher utilization among adults with ADHD does not necessarily reflect greater optimism regarding anticipated benefits.

Similarly, favorable experiences with CHAs were not more common among adults with ADHD after accounting for demographic and socioeconomic factors. In unadjusted analyses, adults with ADHD were less likely to report favorable experiences across several categories [21]. Although these associations were attenuated in adjusted models, point estimates continued to suggest comparable or lower likelihood of positive experiences. This pattern indicates that CHA use among adults with ADHD may reflect ongoing experimentation rather than sustained satisfaction or perceived benefit.

From a community mental health perspective, the divergence between utilization and subjective appraisal is notable. Adults with ADHD may engage in complementary approaches as part of a broader search for symptom relief, particularly in the context of persistent impairment or dissatisfaction with conventional care. These findings do not address clinical effectiveness but instead highlight the complex and iterative nature of health-seeking behavior among adults managing chronic mental health conditions.

Implications for community mental health practice

The high prevalence of CHA use among adults with ADHD has important implications for community mental health practice. Clinicians should be aware that many patients may engage in complementary approaches alongside conventional treatment, often without explicit discussion [9,21,23]. Failure to assess CHA use may contribute to fragmented care, missed opportunities for patient education, or unrecognized interactions with prescribed treatments.

Routine, nonjudgmental inquiry about CHA use may help clinicians better understand patients’ treatment goals, concerns, and experiences. Such conversations can support shared decision-making, promote transparency, and allow clinicians to provide guidance regarding safety and available evidence where appropriate. Acknowledging CHA use does not require endorsement of any specific modalities; rather, it reflects patient-centered care that recognizes real-world behaviors and preferences.

The observed patterns of CHA utilization may also signal unmet needs within existing mental health services for adults with ADHD. Community mental health systems often face constraints related to access, provider availability, and continuity of care. Awareness of CHA use may help clinicians identify areas where patients are seeking support outside the formal system and inform strategies to enhance engagement and satisfaction with evidence-based care.

Policy and systems level implications

At the policy level, increased CHA utilization among adults with ADHD may reflect broader structural challenges in the delivery of adult mental health services. Limited access to specialty ADHD care, variability in insurance coverage, and workforce shortages may contribute to reliance on self-directed or alternative approaches. Understanding these patterns can provide insight into gaps in service provision and areas for system-level improvement. Integrative and collaborative care models may offer opportunities to address some of these challenges by incorporating patient education, behavioral interventions, and coordinated support within existing mental health frameworks. While such models should prioritize evidence-based treatments, awareness of complementary practices may facilitate more comprehensive and responsive care planning. Policymakers and administrators may also consider how insurance coverage, reimbursement policies, and workforce training influence patient reliance on nontraditional care pathways. Finally, population-level-data on CHA utilization can inform public health and educational initiatives aimed at improving health literacy and promoting safe, informed decision-making. Recognizing complementary health approaches as part of real-world care-seeking behavior allows community mental health systems to better align services with patient needs while maintaining appropriate clinical oversight.

Strengths and limitations

This study has several strengths. The use of a nationally representative sample of U.S. adults with over 3,000 respondents enhances generalizability to the adult population and provides a robust foundation for examining CHA utilization among individuals with ADHD. ADHD status was based on self-reported clinician diagnosis and may be subject to recall or reporting bias, including potential misclassification. The focus on adult ADHD addresses an important gap in community mental health research. Additionally, the analytic approach incorporated multiple sociodemographic covariates, reducing the likelihood that observed associations were driven solely by socioeconomic differences.

Several limitations should be considered. The cross-sectional design precludes inference regarding causality or temporal relationships between ADHD diagnosis and CHA use. ADHD status and CHA utilization were assessed by self-report and may be subject to recall or reporting bias. Additionally, the survey did not collect information regarding the type of healthcare professional who made the ADHD diagnosis, the diagnostic methods used, or whether diagnoses were confirmed through standardized clinical assessment. Consequently, we were unable to evaluate potential overdiagnosis, diagnostic misclassification, or variation in diagnostic practices across providers. The survey did not capture ADHD symptom severity, treatment history, medication use, or details on diagnostic methods, nor did it assess the frequency or duration of use of complementary health approaches or associated costs, limiting interpretation of clinical relevance. Favorable expectations and experiences reflect subjective perceptions rather than objective outcomes and should therefore be interpreted cautiously. The proportion of participants reporting an ADHD diagnosis in this sample (15.7%) was substantially higher than commonly cited population-based estimates of adult ADHD prevalence in the United States. This discrepancy may reflect selection bias associated with online opt-in survey panels, increased participation among individuals with mental health conditions, or over-reporting related to self-reported clinician diagnosis. As a result, the findings should not be interpreted as prevalence estimates representative of the U.S. adult population. Although demographic quota controls were applied and adjusted regression models accounted for multiple sociodemographic variables, the elevated prevalence of ADHD in the analytic sample may limit external validity and generalizability of observed associations. Accordingly, these findings should be interpreted as reflecting patterns of complementary health approach utilization within this survey sample rather than definitive population-level estimates. Additionally, the survey did not collect information regarding the type of healthcare professional who made the ADHD diagnosis, the diagnostic methods used, ADHD medication use, treatment history, or treatment response. Consequently, we were unable to evaluate potential overdiagnosis, diagnostic misclassification, whether self-reported diagnoses were corroborated by treatment patterns, or variation in diagnostic practices across providers. The survey also did not assess participants’ experiences with conventional ADHD treatments, including stimulant or non-stimulant medications. Consequently, we were unable to compare favorable expectations or experiences associated with complementary health approaches against perceptions of evidence-based pharmacologic therapies. The survey did not collect information on ADHD pharmacologic treatment, including stimulant or non-stimulant medication use, treatment adherence, or treatment effectiveness. Therefore, we were unable to assess whether complementary health approach utilization was associated with treatment adequacy or satisfaction with conventional ADHD care. Favorable expectations and experiences reflect subjective perceptions rather than objective outcomes and should therefore be interpreted cautiously.

Future research directions

Future research should examine longitudinal patterns of CHA use among adults with ADHD. Prospective studies could clarify whether CHA use precedes, follows, or co-occurs with changes in symptom burden, treatment engagement, or functional outcomes. Qualitative studies may further illuminate patients' motivations, expectations, and decision-making processes related to complementary approaches, particularly in the context of unmet needs. Research examining how CHAs intersect with evidence-based ADHD treatments within community mental health settings is also warranted. Studies evaluating integrative care models, patient education interventions, and clinician-patient communication regarding CHA use may inform strategies to improve care coordination and patient-centered outcomes, particularly in systems seeking to address gaps in adult ADHD services. Future research should also examine whether patterns of complementary health approach utilization observed among adults with ADHD differ from those seen in other psychiatric and neurodevelopmental populations. Comparative studies involving individuals with conditions such as anxiety disorders, depressive disorders, autism spectrum disorder, and other neurodevelopmental conditions may help determine whether elevated CHA utilization reflects ADHD-specific factors or broader patterns of health-seeking behavior among individuals with chronic mental health needs. Such work could improve understanding of disorder-specific and transdiagnostic influences on the use of complementary health approaches. Future research should consider the use of more clinically specific comparator groups when examining complementary health approach utilization. The present study compared adults with self-reported ADHD to adults without ADHD; however, the latter group likely includes individuals with a range of other psychiatric and neurodevelopmental conditions. As a result, the current design does not allow for direct comparison between ADHD and other diagnostic categories such as anxiety disorders, depressive disorders, or autism spectrum disorder. Studies incorporating diagnostic-specific comparison groups would help clarify whether elevated complementary health approach utilization is unique to ADHD or reflects broader patterns among individuals with mental health or neurodevelopmental conditions.

## Conclusions

In this nationally representative sample, adults with attention deficit/hyperactivity disorder (ADHD) reported higher odds of complementary health approach (CHA) utilization than adults without ADHD, after adjusting for demographic and socioeconomic factors. Utilization was most strongly associated with physical and combined psychological and physical approaches, whereas favorable expectations and experiences did not consistently differ by ADHD status. These findings suggest that CHA use among adults with ADHD reflects patterns of care-seeking behavior and potential unmet needs rather than uniformly positive subjective appraisal.

From a community mental health perspective, recognition of CHA use is essential for understanding real-world treatment behaviors among adults with ADHD. Awareness of these patterns may support more comprehensive assessment, enhance clinician-patient communication, and inform efforts to deliver coordinated, patient-centered care. As adult ADHD continues to pose challenges for community mental health systems, understanding how individuals navigate both conventional and complementary care pathways remains an important priority for research and clinical practice.
